# Comparative Study of the Physicochemical, Functional, Thermal, and Rheological Properties of Pretreated Flours From *Dioscorea alata* and *Dioscorea rotundata*

**DOI:** 10.1002/fsn3.71642

**Published:** 2026-03-23

**Authors:** Piedad Montero Castillo, Diofanor Acevedo Correa, Karina Vivanco Zuñiga

**Affiliations:** ^1^ Faculty of Engineering, Working in the Agricultural and Agroindustrial Innovation and Development Research Group Universidad de Cartagena Cartagena Colombia; ^2^ Faculty of Economic Sciences, Working in the Innovation, Administration and Engineering Research Group Universidad de Cartagena Cartagena Colombia

**Keywords:** blanching, Dioscorea flour, food industry, functional properties, immersion, soaking

## Abstract

*Dioscorea* is a diverse group of tubers known as yams, among which *D. alata* and *D. rotundata* stand out for their socioeconomic importance. For preservation purposes, they are processed into flours, which may present technological limitations. Pretreatments such as immersion, blanching, and soaking have shown potential for improving their quality. In this context, the objective of this research was to conduct a comparative study of the physicochemical, functional, thermal, and rheological characteristics of yam flours from *D. alata* and *D. rotundata* subjected to pretreatments. Flours from both varieties were obtained by immersing them in 1.5% citric acid for 10 min, blanching them at 50°C for 3 min, and soaking them in water for 4 h. Physicochemical analysis revealed that pretreatments improve the whiteness index, while proximal analysis indicated a higher carbohydrate content. Improvements in functional properties were evident, with a retention capacity of 2.98 g/g for *D. alata* and a solubility and dispersibility of 9.24% and 70%, respectively, for *D. rotundata* . Thermally, the flours had gelatinization temperatures above 70°C and a degradation limit of 200°C, conditions suitable for cooking processes. The rheological behavior was typical of shear thinning and G´ > G´´ modules, indicating viscoelastic solid behavior, although *D. alata* showed the highest resistance to flow. In general, the flours presented varied characteristics that allow decisions to be made in the selection and optimization of processes and products in the food industry.

## Graphical abstract

Pretreatments help improve flour quality. The increase in solubility and dispersibility is greater in *D. rotundata. D. alata* flours are more resistant to shear than *D. rotundata* flours. All flours degrade after 200°C. *D. alata* flours are more elastic while *D. rotundata* flours are more viscous.

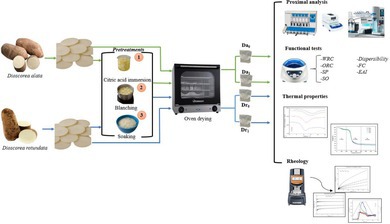

## 1 Introduction

*Dioscorea* species, generally known as “yams,” belong to the *Dioscoreaceae* family (Sugihara et al. 2021) composed of about 600 species distributed in Africa, Asia, the Caribbean, Oceania, and Latin America (Fawale et al. 2025; Wang et al. 2024). It has been documented that between 10 (Lebot et al. 2024) and 11 yam species are cultivated and considered food crops, *D. dumetorum*, *D. bulbifera*, *D. esculenta*, *D. nummularia*, *D. oppositifolia*, *D. polystachya*, *D. pentaphylla*, *D. trifida*, *D. cayenensis*, *D. alata*, *and D. rotundata*; however, only the last three are considered important food crops due to its increased production and spread across different regions of the world (Lawal et al. 2023; Lebot et al. 2023).

Currently, yams are the world's fourth most widely produced edible tuber and rhizome crop, after potatoes, cassava, and sweet potatoes (Zang et al. 2024), with global production reaching 1.55 billion tonnes in 2023 (FAO 2023). In Colombia, yam production reached 231.5 thousand tonnes in 2024 (Agronet 2024) and, in line with the world trend, the species *D. alata* and *D. rotundata* are the most widely cultivated (Lebot et al. 2024; Mercado et al. 2025). The Caribbean region is the main yam‐producing area in the country (Guillermo et al. 2021), with production concentrated in the departments of Córdoba, Sucre, and Bolívar, which together account for 83.4% of national production (Agronet 2024). However, the yam production chain in Bolívar has focused mainly on marketing the fresh product (Suárez et al. 2023).

In this sense, it is important to highlight the main properties of yam as a food material that stands out for being rich in nutritional and functional components (Lawal et al. 2023) that include proteins, fats and starchy carbohydrates (Argaw et al. 2023), however, due to its perishable nature mediated by its high moisture content, it has a short shelf life under environmental conditions (Datir et al. 2024). In this sense, the transformation of yam into flour has emerged as a conservation strategy (Argaw et al. 2023). Moreover, due to its versatility of uses, it is an ideal raw material for food applications (Argaw et al. 2023). Recent studies have explored the use of *D. alata* (Awoyale et al. 2024; Huang, Cheng, et al. 2024; Naimah et al. 2023) and *D. rotundata* (Ikegwu et al. 2025; Ufondu et al. 2022) flours in foods, showing favorable results, which is evidence of the great impact that these flours can have in the food industry.

It should be noted that, although yam has a high nutrient content, it also contains compounds that limit its processing, such as peroxidase and polyphenoloxidase enzymes, which are responsible for rapid enzymatic browning (Eshun et al. 2022) after damage from cutting and exposure to oxygen (Huang, Cheng, et al. 2024). In response to this limitation, previous research has evaluated techniques such as the use of citric acid treatment between 1% and 5% to inactivate these enzymes, improve sensory attributes (Amedor, Sarpong, et al. 2024; Pang et al. 2024), and microbiological quality (Amedor, Owusu‐Kwarteng, et al. 2024). In addition, blanching has been studied as a complementary hydrothermal treatment to immersion. This rapid heat treatment can modify sensory attributes and functional properties in flours (Rodríguez‐Lora et al. 2022). Blanching conditions between 50°C and 100°C have been evaluated (Amedor, Sarpong, et al. 2024; Uthayakumaran et al. 2025); however, studies on *D. esculenta* and tubers such as yacon suggest implementing temperatures below 60°C (Simatupang and Witdarko 2021).

On the other hand, it is important to note that *Dioscorea* tubers contain significant amounts of undesirable anti‐nutritional factors (Lawal et al. 2023) that can cause damage to health (Sahoo et al. 2025) and bitter taste (Adebowale et al. 2018). These compounds are easily removed by conventional processing techniques, such as soaking, due to their solubility in water (Tareen et al. 2025). In addition, it has been observed that this detoxification technique can modify functional characteristics, potentially altering the water and oil absorption capacity and swelling power of flours due to the changes it generates in their chemical composition (Estiasih et al. 2022). Recently, Sahoo et al. (2025) explored soaking for 4 h and found that a significant reduction in the content of anti‐nutritional factors was achieved; however, longer soaking periods have been associated with significant nutrient loss (Quayson et al. 2021).

From a more general perspective, the advantages of immersion, blanching, and soaking in the processing of yams into flour have been demonstrated by previous research. This research proposes the joint application of these pretreatments to inactivate unwanted components such as peroxidase and polyphenoloxidase and antinutrients, as there is a gap in the literature regarding the joint evaluation of these pretreatments. Therefore, this study represents the first study to evaluate its comprehensive application by exploring how it affects the quality of flours from *D. alata* and *D. rotundata* , two of the most widely cultivated species in the world and particularly in Colombia. Furthermore, despite the importance of these crops, available studies have focused mainly on isolated assessments of their techno‐functional properties, with limited information on comparative analyses of these varieties.

Therefore, the objective of this research was to carry out a comparative study of the physicochemical, functional, thermal, and rheological characteristics of *Dioscorea alata* and *Dioscorea rotundata* yam flours subjected to pretreatments.

## 2 Materials and Methods

For the study, yams of the varieties *D. alata* and *D. rotundata* from the village of San Cayetano, in the municipality of San Juan Nepomuceno, subregion of Montes de María, Bolívar, were used. Initially, yams were selected and inspected for signs of deterioration according to Colombian technical standard NTC 1269: 1976, until reaching an approximate weight of 50 kg of each variety.

For proximal analyses and the determination of total starch and amylose content, sulfuric acid (95%), sodium hydroxide, hydrochloric acid (30%, A.R.), petroleum ether, boric acid, phenolphthalein, anthrone, and iodine (I_2_ ≥ 99%) were used. All reagents were of analytical grade and supplied by Elementos Químicos Ltda. (Bogotá, Colombia).

### 2.1 Flour Processing and Study Design

The yams were washed with clean water to remove dirt and unwanted particles, then peeled and cut into 2 mm‐thick slices. Subsequently, the slices of *D. alata* and *D. rotundata* were subjected to a series of treatments in the following order: immersion in 1.5% citric acid for 10 min (Eshun et al. 2022), blanching at 50°C for 3 min (Obadina et al. 2014), and soaking in water at room temperature for 4 h (Abiodun et al. 2009), following the methodologies proposed by the authors. Finally, the slices were dried at 70°C for 2 h in a forced convection oven (Tornado, Colombia), ground, and sieved through a N60 mesh until a fine powder was obtained. Pre‐treated flours of *D. alata* and *D. rotundata* were obtained and identified as *Da*_*1*_ and *Dr*_*1*_, respectively. The samples were packed in airtight metallized bags.

On the other hand, control samples of each variety were prepared without pretreatments, which were dried and packaged under the same conditions previously mentioned. In this sense, control *D. alata* yam flour, *Da*_*0*_, and control *D. rotundata* yam flour, *Dr*_*0*_, were obtained.

For each sample, triplicate analyses were performed based on the following tests:

### 2.2 Physicochemical Characterization

#### 2.2.1 Determination of pH and Acidity

The pH measurement was performed using the method described by Ngoma et al. (2019) using a pH meter (Hanna Instruments, Italy), previously calibrated with buffer solutions of pH 4, 7, and 10. Acidity was determined by titration with NaOH.

#### 2.2.2 Determination of Color

Color measurements of flour samples were carried out following the method described by Kimbonguila et al. (2019) and Setyawan et al. (2021), using a colorimeter (Konica Minolta, Colombia). This equipment was adjusted to the *L*, *a*, and *b* scales. The whiteness index (WI) was calculated according to the following equation:

(1) $$\mathrm{WI}=\sqrt{{\left(100-L\right)}^{2}+a^{2}+b^{2}}$$

#### 2.2.3 Proximal Analysis

The proximate analysis of the yam flours was carried out using the methods standardized by AOAC (2016). Moisture content was determined by weight difference; fat by the Soxhlet method; fiber by acid and basic hydrolysis; ash by muffle incineration; and protein by the Kjeldahl method, using equipment based on these methods. Carbohydrate content was determined by difference. The gross energy (kJ/100 g^−1^) of yam flour was calculated by the procedure described by Sahoo et al. (2023).

#### 2.2.4 Total Starch, Amylose, and Amylopectin Content

Starch content was determined by the anthrone reagent method, amylose content was determined using the iodine binding method, both as reported by Sahoo et al. (2023). On the other hand, amylopectin content was determined by difference.

#### 2.2.5 Particle Size Distribution

The size distribution of the flour granules was determined following the method described by Xiao et al. (2023) with a laser particle size analyzer (Mastersizer 3000; Malvern Panalytical, England). The volume, surface area, and numerical distribution of the flour granules were determined. Particle size distributions at D [10], D [50], D [90], D [4;3], and D [3;2] were automatically calculated with Mastersizer 3000 software.

#### 2.2.6 Determination of Loose Bulk Density and Packed Bulk Density

The loose bulk density was determined according to the method described by Kayode et al. (2023). A 100 mL measuring cylinder was filled with 50 g of yam flour, and the volume occupied by the sample was recorded. On the other hand, the packed bulk density was determined by using the method described by Ngoma et al. (2019). Fifty‐gram samples were taken and placed in a 100 mL graduated measuring cylinder. The cylinder was then tapped several times on a laboratory bench until a constant volume was reached. The loose and packed bulk densities were calculated by dividing the flour weight (in grams) by the volume (mL).

### 2.3 Functional Properties

#### 2.3.1 Water Retention Capacity and Oil Retention Capacity

The determination of this property was carried out following the methodology described by Kayode et al. (2023). To determine the water absorption capacity, 10 mL of distilled water was added to 0.5 g of flour at room temperature. The mixture was then incubated in a water bath at 60°C for 30 min and subsequently centrifuged at 3000 rpm for 15 min. Then, the sediment tubes were weighed and based on Equation (2), the water retention capacity (WRC) was established.

(2) WRCgg=C2−C1H

where $\begin{array}{c} C_{2}=\text{Weight of tube with sediment};C_{1}=\mathrm{ml}\;\text{of distilled water};\text{and}\\ H=\text{sample weight.} \end{array}$

The oil absorption capacity (ORC) was determined using the modified method of Argaw et al. (2023). 0.5 g of sample was weighed into a pre‐weighed centrifuge tube, and 6 mL of sunflower oil was added. The mixture was incubated at 25°C for 30 min and then centrifuged at 5000 rpm for 30 min. Excess oil was drained off, and the tube was placed (inverted) on absorbent paper for 25 min to remove excess oil. The density of the sunflower oil was taken as 0.918 g/mL. The final weight of the tube was determined, and the oil retention capacity (ORC) of the flour was calculated using the following equation:

(3) ORCmlg=Tf−H−T0/dH

where $\begin{array}{c} T_{f}=\text{Weight of tube with sediment};H=\text{Flour sample weight};T_{0}=\\ \text{Empty tube weight};\text{and}\;d=\text{density of sunflower oil}. \end{array}$

#### 2.3.2 Swelling Power and Solubility

Swelling power (SP) and solubility (S) were determined following the method described by Argaw et al. (2023). Approximately 0.2 g of yam flour was weighed and 15 mL of distilled water was added into a centrifuge tube which was then immersed in a water bath at a temperature of 85°C for 30 min with constant stirring. The tube was then removed, cooled to room temperature, and centrifuged at 6000 rpm for 15 min. The upper solution of each sample was transferred to an evaporation plate and dried at 110°C for 3 h. Finally, both the residue representing the amount of sample dissolved in water and the insoluble sample were weighed. SP and S were calculated using the equations given below:

(4) SPgg=sediment weightsample weight−soluble weight

(5) $$\%S=\frac{\text{soluble weight}}{\text{sample weight}}$$

#### 2.3.3 Dispersability

Dispersibility was determined following the method described by Oyeyinka et al. (2023). Initially, it was measured by weighing 10 g of each sample in a 100 mL measuring cylinder and distilled water was added until the 100 mL mark was reached. The setup was shaken and allowed to stand for 3 h. The volume of settled particles was recorded and subtracted from 100. The difference was reported as percent dispersibility.

#### 2.3.4 Foaming Capacity

The foaming capacity (FC) of tuber flour was determined as a percentage, following the methodology described by Hu et al. (2018) and by Ijabadeniyi et al. (2023) with slight modifications. For this purpose, 3 g of yam flour was transferred to 50 mL cylinders, to which 30 mL of distilled water was added, mixed, and the initial volume was recorded. The mixture was then shaken vigorously, and the initial volume was recorded, considering the volume of bubbles. The CFE was expressed as a percentage of foam formed.

#### 2.3.5 Emulsion Activity Index

The emulsion activity index (EAI) of the flours was determined as described by Ijabadeniyi et al. (2023). 5 mL of distilled water and 5 mL of sunflower oil were added to 50 mg of the flour sample. The mixture was shaken for 3 min and then centrifuged at 1100 rpm for 5 min. The height of the emulsifying layer was recorded, and the IAE was determined as shown in Equation (6).

(6) $$\mathrm{EAI}\left(\%\right)=\frac{\text{Emulsified layer height}}{\text{Total height}}*100.$$

### 2.4 Thermal Properties

The thermal properties of the yam flours were measured following the procedure described by Argaw et al. (2024) with minor modifications, using a differential scanning calorimeter (DSC Q 2000, TA Instruments). 3 mg of each yam flour was weighed into a hermetically sealed aluminum capsule, and distilled water was added in a 1:4 ratio and allowed to stand for 4 h. For measurements, an empty capsule was used as a reference. The samples were heated at a rate of 10°C/min up to 120°C. Onset (To), peak (Tp), end (Tc), and gelatinization enthalpy (ΔH·J/g dry weight) temperatures were measured.

### 2.5 Thermogravimetric Analysis

Thermogravimetric analyses of the yam flours were carried out on a Linseis STA PT1600 balance following the method described by Sahoo et al. (2023). Samples were heated in alumina crucibles from room temperature to 800°C at a heating rate of 10°C/min and a nitrogen flow rate of 50 mL/min, in an inert atmosphere.

### 2.6 Rheological Characteristics of Yam Flour and Pasting Properties

The rheological characteristics of the yam flour were evaluated using a rotational rheometer (HR 10 Discovery, TA Instruments) following the method described by Argaw et al. (2024). Suspensions of 5% yam flour in distilled water were prepared by heating it in a water bath at 90°C for 7 min and allowing it to cool to room temperature. A 40 mm stainless steel parallel plate with 1 mm spacing was used. A constant shear test was performed to obtain flow curves in the range of 1–1000 s^−1^ at 25°C. These were fitted to the Heschel Bulkley model, described by the following equation:

(7) $$\sigma =\sigma_{0}+K{\dot{\gamma }}^{n},$$

where σ is the shear stress $\left(\mathrm{Pa}\right)$, $\sigma_{0}$ the yield stress $\left(\mathrm{Pa}\right)$, K the consistency coefficient $\left(\mathrm{Pa}.s^{n}\right)$, $\dot{\gamma }$ the shear velocity $\left(s^{-1}\right)$, and n the flow behavior index (Martínez‐Padilla 2022).

On the other hand, the gelatinized sample was subjected to tensile scanning to obtain the elastic modulus (G′) and viscous modulus (G″) and loss factor (tan δ). Measurements were recorded over a range of 0.1–100 rad/s with a constant strain of 0.2% at 25°C.

The pasting properties of the flours were determined based on the method described by Rodríguez‐Lora et al. (2022). Flour suspensions were prepared at 5% (w/w), homogenized, and transferred to the Peltier plate of the rheometer. Subsequently, a temperature sweep was applied, which consisted of maintaining the sample at 50°C for 1 min, followed by an increase in temperature at a rate of 6°C/min until it reached 95°C; it was then maintained at this temperature for 5 min. Next, the sample was cooled at the same rate to 50°C and held for 2 min. The tests were performed in triplicate, and the values of paste temperature, paste time, peak viscosity, minimum viscosity, breakdown, final viscosity, and setback were recorded.

### 2.7 Data Analysis

All data from control and pretreated samples were analyzed using Statgraphics Centurion software (Statgraphics Technologies Inc., USA). Means of three replicates were used for statistical analyses. Analysis of variance (ANOVA) and means were calculated using Tukey's multiple comparisons test at 95% confidence (*p* < 0.05).

## 3 Results and Discussion

### 3.1 Physicochemical Properties

Table 1 shows the results of the physicochemical characterization of *D. alata* and *D. rotundata* flours. The pH of the *D. alata* samples showed slightly acidic values, especially in the sample with *Da*_*1*_ pretreatment, exhibiting significant differences. This behavior was also observed in the *D. rotundata* flours, which have been attributed mainly to the immersion in citric acid, which is considered an acidifying agent (Tamaroh and Purwani 2021). The pH values shown in Table 1 are within the ranges reported by Tanimola et al. (2022), who reported pH values between 5.59 and 7.71 in starchy materials obtained from *D. alata* , and between 6.29 and 7.26 in *D. rotundata* . It is important to note that, as reported by Tortoe et al. (2017) and Bikila et al. (2022), flours should ideally have pH values above 6, since it is considered that at this pH flours do not have a sour taste, can show little or no starch breakdown, and are desirable and suitable for a wide range of dietary applications, including baking. In this regard, *D. rotundata* flours (*Dr*_*0*_ and *Dr*_*1*_) are considered suitable.

**TABLE 1:** pH, titratable acidity, and color in flours of *D. alata* and *D. rotundata* yams.

Analysis/sample	*D. alata*		*D. rotundata*	
	*Da*_*0*_	*Da*_*1*_	*Dr*_*0*_	*Dr*_*1*_
pH	5,93 ± 0,14 ^b^	5,63 ± 0,15 ^c^	6,26 ± 0,03 ^a^	6,06 ± 0,02 ^ab^
Acidity	0,56 ± 0,01 ^b^	0,64 ± 0,02 ^a^	0,49 ± 0,01 ^c^	0,44 ± 0,01 ^d^
L*	91,01 ± 0,03 ^a^	90,31 ± 0,04 ^b^	89,55 ± 0,17 ^b^	90,15 ± 0,19 ^c^
a	‐0,44 ± 0,02 ^b^	‐0,15 ± 0,03 ^a^	‐0,84 ± 0,02 ^c^	‐1,08 ± 0,04 ^d^
b	9,91 ± 0,11 ^a^	7,40 ± 0,08 ^d^	9,09 ± 0,08 ^b^	8,43 ± 0,14 ^c^
%WI	86,61 ± 0,10 ^b^	87,80 ± 0,07 ^a^	86,12 ± 0,17 ^c^	86,98 ± 0,18 ^b^

*Note:* Means with the same letter in the same row are not significantly different (*p* < 0.05).

The titratable acidity of the pretreated flour of *D. alata* showed higher values than the control sample, due to the absorption of hydrogen ions during immersion in citric acid. However, in *D. rotundata* , this pattern was not observed, since the control sample, despite having a more basic pH, presented a higher acidity value. Quayson et al. (2021) reported similar behavior in soaked yams of *D. alata* and *D. rotundata* , with nonlinear acidity trends, and pointed out that this trend occurs in most studies that include soaking and was attributed to the limited interaction between water and yam slices being a heterogeneous system.

The results of the colorimetric analysis indicated that there are significant differences in the L* value of the treated and untreated samples. The *D. alata* flours exhibited a higher lightness compared to the *D. rotundata* flours, which suggests that these samples appear whiter. The values were within the range reported by Kimbonguila et al. (2019) who reported L* values between 84.82 and 91.49 for *D. alata* , and Amankwah et al. (2022) who reported values in the range of 76.37 and 90.26 for *D. rotundata* , thus confirming our findings. It is important to note that, in *D. alata* samples, a reduction of the L* value was observed similar to that reported by Eshun et al. (2022) who observed that, although immersion in citric acid can decrease the activity of enzyme peroxidase and polyphenol oxidase, responsible for the loss of brightness, minimal browning reactions can still occur due to other factors, such as phenolic content. In addition, it has been reported that the yam *D. rotundata* has a higher polyphenol content than *D. alata* (Lombe et al. 2023), which makes it more sensitive to oxidation processes and, therefore, to color changes, which could justify the difference between the L* values.

The parameter *a* ranged from −1.08 to −0.15, indicating green hues (Adeboyejo et al. 2021), although statistically significant differences were observed (*p* < 0.05) did not reach a sufficiently high threshold of perception to differentiate *D. rotundata* cultivars from *D. alata* , based on this index alone, in line with that reported by Tortoe et al. (2017). As for parameter b, a decrease is observed in the pretreated flours compared to the controls. According to Ratnaningsih et al. (2018), acid immersion, soaking (Abiodun et al. 2013; Krishnan et al. 2010), and blanching (Desalegn and Olika 2022) contribute to the reduction of browning in the flours. Consequently, this decrease was reflected in the high %WI values of the samples, However, the reduction of browning has not been attributed in the literature exclusively to the inhibition of enzymatic activities, but also to the loss of phenolic compounds during blanching (Setyawan et al. 2021) and drying (Uthayakumaran et al. 2025).

#### 3.1.1 Proximal Analysis, Starch, Amylose, and Amylopectin Content

The proximate analysis of *D. alata* and *D. rotundata* flours is presented in Table 2.

**TABLE 2:** Proximal composition and total starch, amylose, and amylopectin content of *D. alata* and *D. rotundata* yam flours.

Analysis/sample	*D. alata*		*D. rotundata*	
	*Da*_*0*_	*Da*_*1*_	*Dr*_*0*_	*Dr*_*1*_
Moisture (%)	8,92 ± 0,17 ^a^	6,10 ± 0,25 ^b^	5,84 ± 0,36 ^b^	4,62 ± 0,60 ^c^
Fat (%)	0,40 ± 0,08 ^b^	0,35 ± 0,01 ^b^	0,52 ± 0,10 ^a^	0,45 ± 0,14 ^ab^
Crude fiber (%)	1,60 ± 0,04 ^b^	1,50 ± 0,03 ^bc^	2,11 ± 0,04 ^a^	1,45 ± 0,03 ^c^
Ashes (%)	2,59 ± 0,06 ^a^	1,80 ± 0,01 ^b^	1,49 ± 0,02 ^c^	0,64 ± 0,03 ^d^
Protein (%)	6,06 ± 0,35 ^a^	5,71 ± 0,05 ^a^	4,66 ± 0,13 ^b^	3,93 ± 0,08 ^c^
Carbohydrates (%)	82,01 ± 0,67 ^c^	86,01 ± 0,32 ^b^	87,46 ± 0,62 ^b^	90,33 ± 0,86 ^a^
GE $\left(\mathrm{KJ}*100g^{-1}\;\mathrm{MS}\right)$	1512,28 ± 2,51 ^c^	1572,63 ± 4,41 ^b^	1573,25 ± 5,34 ^b^	1631,95 ± 7,01 ^a^
Total starch (g/100 g)	82,60 ± 0,76 ^b^	87,02 ± 0.52 ^a^	83,21 ± 0,45 ^b^	87,15 ± 0,52 ^a^
Amylose (g/100 g)	30,74 ± 0,55 ^b^	34,07 ± 0,84 ^a^	12,42 ± 0,71 ^d^	22,14 ± 0,62 ^c^
Amylopectin (g/100 g)	69,26 ± 0,55 ^c^	65,93 ± 0,84 ^d^	87,58 ± 0,71 ^a^	77,86 ± 0,62 ^b^

*Note:* Means with the same letter in the same row are not significantly different (*p* < 0.05).

The moisture of *Da*_*1*_ and *Dr*_*1*_ samples decreased with the implemented pretreatments, similar to previous studies on *D. rotundata* (Amedor, Owusu‐Kwarteng, et al. 2024; Amedor, Sarpong, et al. 2024) and *D. alata* (Uthayakumaran et al. 2025). It has been reported that blanching improves drying kinetics by modifying the internal structure, which increases porosity and facilitates water movement (Uthayakumaran et al. 2025), implying that blanching pretreatment decreased the amount of energy needed to initiate mass diffusion from inside the slices during the drying process (Taiwo et al. 2021). Similarly, citric acid treatment has been shown to increase the permeability of cell membranes by softening the tissues (Amedor, Sarpong, et al. 2024), which justifies our findings.

The fat, ash, fiber, and protein content showed a decrease in the pretreated samples. In general terms, and according to Alenyorege et al. (2024) and Kumoro et al. (2020), pretreatment, soaking, and bleaching can cause nutrient loss due to the leaching of compounds into the water, which explains the decreases observed. It should be noted that the reason for the decrease in protein content of treated flours compared to native flours is attributed to protein denaturation (Uthayakumaran et al. 2025).

The reduction of these components, together with the decrease in moisture, concentrated carbohydrates, significantly increasing their content in the treated samples, whose values were within the ranges reported by Obadina et al. (2014) and Argaw et al. (2023) in flours of *D. rotundata* and *D. alata* , respectively. Consequently, GE increased in all samples. The GE values of the samples are similar to those reported in the literature (Omohimi et al. 2018; Sahoo et al. 2023) and comparable with data reported for wheat flour (Liu et al. 2018), which underlines the use of *Dioscorea* flours as an alternative energy source (Setyawan et al. 2021).

Starch is the main carbohydrate present in yam flours (Sahoo et al. 2023); therefore, the increase in carbohydrate content was reflected in a higher total starch content in the treated samples *Da*_*1*_ and *Dr*_*1*_. In addition, an increase in amylose content was observed. This phenomenon has been attributed to the rupture of some amylopectin branches, which envelop the amylose, under the hydrothermal effect, leading to the formation of new amylose chains, which, in turn, bind to the original amylose by hydrogen bonds, thus reducing the amylopectin portion (Desai et al. 2024; Mowafy et al. 2024). Similarly, treatment with citric acid increases amylose due to acid hydrolysis of amylopectin molecules (Shrivastava et al. 2024).

Based on the amylose content of starches, a classification has been established for starchy materials, categorizing them as: waxy (0%–5%), very low in amylose (5%–12%), low in amylose (12%–20%), intermediate amylose (20%–25%), or high in amylose (25%–33% or more) (Sahoo et al. 2023). Based on this information and according to Table 2, it can be established that the flours of *D. alata* , *Da*_*0*_ and *Da*_*1*_, are high in amylose, while those of *D. rotundata* , *Dr*_*0*_ and *Dr*_*1*_, are intermediate in amylose.

Importantly, it has been shown that amylose content correlates significantly with lower digestibility and higher resistant starch content (Yu et al. 2021). Therefore, its increase can provide a functional benefit to consumers by promoting a more delayed digestion of carbohydrates (Zang et al. 2024), which makes these flours a suitable option for the production of functional foods such as high‐fiber breads and crackers (Tekin and Fisunoglu 2023; Falsafi et al. 2022), low‐fat meat products (Wang et al. 2025), and low‐calorie dairy products (Karunarathna et al. 2024).

#### 3.1.2 Particle Size Distribution and Bulk Density

**FIGURE 1:**
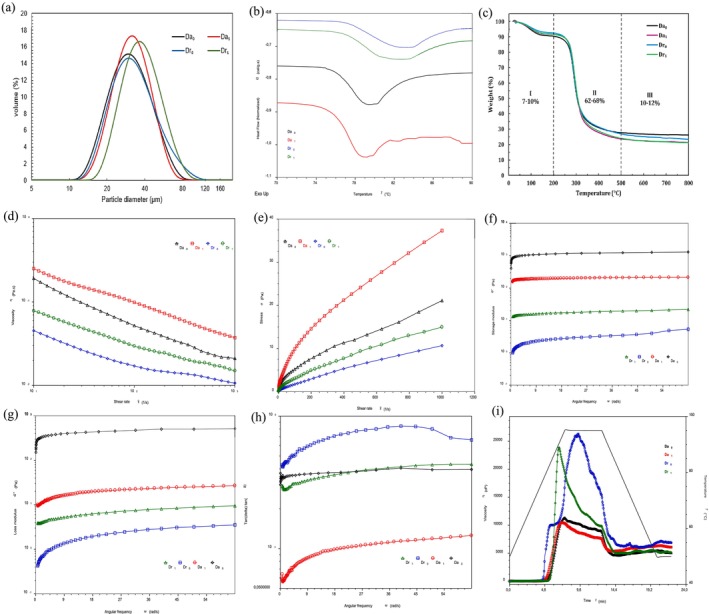
(a) Particle size distribution of yam flours; (b) DSC thermogram of pretreated and non‐pretreated *D. alata* and *D. rotundata* flours; (c) T_g_ curves and weight reduction of pretreated and untreated *D. alata* and *D. rotundata* yam flours; (d) Rheogram of *D. alata* and *D. rotundata* flours adjusted to the Herschel Bulkley model; (e) Apparent viscosity vs. shear rate in pretreated and non‐pretreated flours; (f) Storage modulus of yam flours; (g) Loss modulus of yam flours; (h) Tan of yam flours; (i) Pasting properties of pretreated and non‐pretreated *D. alata* and *D. rotundata* flours.

The particle size distribution obtained by laser diffraction of the different yam flours is presented in Figure 1a. The flours of *D. alata* and *D. rotundata* exhibited a monomodal distribution, single‐peaked curves, with a diameter distribution range of 9.24–118.92, 9.24–104.58, 9.24–153.38, and 11.93–134.99 μm, for *Da*_*0*_, *Da*_*1*_, *Dr*_*0*_, and *Dr*_*1*_, respectively.

In Table 3, the parameters D [10], D [50] and D [90] are presented, which indicate the particle sizes below which 10%, 50%, and 90% of the total volume is found; these values were in the range of 18.8–24.13, 30.21–37.16, 48.51–59.13 μm, respectively, which coincided with the range reported by Ye et al. (2024). The trends of changes in the parameters D [10] and D [90] were similar to D [50], except D [90] in sample *Da*_*1*_, where the mean value decreased without exhibiting significant differences. Nevertheless, D [50] is considered the most representative parameter to describe the particle size distribution, by dividing the sample into two equivalent halves (Cristiano et al. 2019). An increase in D [50] was evidenced with the application of the pretreatments in samples *Da*_*1*_ and *Dr*_*1*_, whose values were found within the ranges reported by Wu et al. (2022). This behavior was also observed by Badiora et al. (2023) in sweet potato flours.

**TABLE 3:** Particle size distribution and bulk density parameters of *D. alata* and *D. rotundata* yam flours.

Analysis/sample	*D. alata*		*D. rotundata*	
	*Da*_*0*_	*Da*_*1*_	*Dr*_*0*_	*Dr*_*1*_
D [10] μm	18,80 ± 0,47 ^c^	20,45 ± 1,34 ^b^	19,60 ± 0,68 ^bc^	24,13 ± 0,63 ^a^
D [50] μm	30,21 ± 0,66 ^c^	31,48 ± 0,82 ^b^	31,71 ± 0,59 ^b^	37,16 ± 0,39 ^a^
D [90] μm	49,98 ± 0,79 ^c^	48,51 ± 1,64 ^c^	56,60 ± 1,52 ^b^	59,13 ± 1,16 ^a^
D [4;3] μm	32,56 ± 0,48 ^c^	33,18 ± 0,57 ^c^	35,43 ± 0,64 ^b^	39,73 ± 0,28 ^a^
D [3;2] μm	28,48 ± 0,53 ^b^	29,86 ± 1,0 ^b^	30,18 ± 0,66 ^b^	36,26 ± 1,78 ^a^
Bulk density loose gml	0,64 ± 0,02 ^ab^	0,66 ± 0,01 ^a^	0,60 ± 0,07 ^c^	0,61 ± 0,01 ^bc^
Bulk density packed gml	0,90 ± 0,00 ^b^	0,85 ± 0,02 ^c^	0,92 ± 0,00 ^a^	0, 84 ±, 008 ^c^

*Note:* Means with the same letter in the same row are not significantly different (*p* < 0.05).

On the other hand, D [4;3] and D [3;2], which represent the volume‐weighted mean particle diameter and surface area‐weighted mean diameter (Chen et al. 2025), also showed increases with pretreatments, the values were within the ranges reported by Ouyang et al. (Ouyang et al. 2024) and Ochoa and Osorio‐Tobón (2024) for each parameter. These results imply that larger particle sizes generate greater diameter and surface area.

Recently, it was found that treatments that increase the amylose portion have been related to an increase in particle size distribution (Almeida et al. 2024). This is because, as amylopectin is broken down into amylose, small granules become looser and more mobile, facilitating their aggregation into larger particles (Ye et al. 2024). In this context, it can be stated that during blanching and immersion in acid, amylopectin degradation generates amylose molecules that tend to aggregate, forming larger agglomerates, which explains the observed behavior.

In addition to particle size distribution, bulk density is a crucial parameter in powdered food handling, as it influences packaging, storage, and processing (Wonglek et al. 2024). The results in Table 3 show that there are no significant differences in the loose bulk density of *D. alata* and *D. rotundata* flours, indicating that pretreatments do not affect this parameter.

The packed bulk density results of *D. alata* and *D. rotundata* samples decreased with the implementation of the pretreatments. Despite the decreases, the values obtained were within the ranges reported for blanched *D. alata* yam flours, 0.82–0.88 g/mL (Adejumo et al. 2013); and for *D. rotundata* , 0.83–0.88 g/mL (Wahab et al. 2016). This behavior was attributed to the increase in particle size. Nevertheless, high values of packed bulk density were obtained, which offer advantages in bulk storage and transport, as it allows a larger volume of packed product within a constant space (Babalola et al. 2021). In addition, yam flours with high packed bulk density can be useful in pastes to reduce thickness (Ngoma et al. 2019).

### 3.2 Functional Properties

*Dioscorea* flours are mainly composed of polysaccharides (Jiang et al. 2024) such as starch (Bora et al. 2023), on which the functional properties depend to a large extent; however, other components such as proteins and fats also play a role (Asranudin et al. 2023). Understanding these properties from an application approach is relevant in food processing (Sahoo et al. 2023). The results of the functional properties are presented in Table 4.

**TABLE 4:** Functional properties of yam flours *D. alata* and *D. rotundata* .

Análisis/Muestra	*D. alata*		*D. rotundata*	
	*Da*_*0*_	*Da*_*1*_	*Dr*_*0*_	*Dr*_*1*_
WRC gg	2,8 ± 0,028 ^b^	2,9 ± 0,057 ^a^	2,44 ± 0,02 ^d^	2,5 ± 0,014 ^c^
ORC gg	1,00 ± 0,01 ^a^	0,92 ± 0,01 ^b^	1,04 ± 0,0 ^a^	1,01 ± 1,01 ^a^
SP gg	6,26 ± 0,13 ^c^	4,65 ± 0,11 ^d^	11,47 ± 0,46 ^a^	8,44 ± 0,22 ^b^
S (%)	8,92 ± 0,45 ^a^	9,24 ± 0,58 ^a^	8,63 ± 0,62 ^a^	9,51 ± 0,75 ^a^
Dispersibility	56,66 ± 0,57 ^c^	64,33 ± 0,57 ^b^	64,33 ± 1,15 ^b^	70,16 ± 0,28 ^a^
FC (%)	5,20 ± 0,90 ^c^	5,1 ± 0,88 ^c^	24,74 ± 1,36 ^a^	12,06 ± 0,27 ^b^
EAI (%)	45, 00 ± 5,00 ^a^	41,66 ± 2,88 ^a^	43,66 ± 2,08 ^b^	40,00 ± 1,00 ^b^

*Note:* Means with the same letter in the same row are not significantly different (*p* < 0.05).

#### 3.2.1 Water Retention Capacity

The WRC is an indicator of the amount of water that a material can absorb and retain about its weight (Tortoe et al. 2017) and is closely related to starch properties (Subroto et al. 2024). In this study, the WRC of the flours was in the range of 2.44–2.98 g/g, similar to that reported by Wu et al. (2022) and significant differences were observed between samples with and without pretreatment of each variety. An increase in WRC was evidenced in the pretreated flours, a behavior previously reported in potato (Asranudin et al. 2023) and sweet potato (Desalegn and Olika 2022) flours, subjected to blanching and immersion in citric acid, respectively. This increase has been associated with a reduction in the content of branched macromolecules (amylopectin) (Mowafy et al. 2024) and a higher amylose content (Amankwah et al. 2022), which favors a greater affinity with water due to its hydrophilic nature (Asranudin et al. 2023). In agreement with the information reported in Table 2, with respect to amylose content, the *Da*_*0*_ and *Da*_*1*_ samples presented the highest WRC values, the latter being the one with the highest capacity. A high WRC is desirable in the formulation of bakery products, processed cheeses, and sausages (Ngoma et al. 2019).

#### 3.2.2 Oil Retention Capacity

In contrast to WRC, ORC exhibited an opposite trend; however, no significant differences were observed among *D. rotundata* flours, but ORC was observed in *D. alata* . ORC has been defined as the ability of flour proteins to bind with oil (Godfrey et al. 2023). As observed by Asranudin et al. (2023), the increase in hydrophobic properties is determined by the protein content of the flour. Consequently, the reduction in protein content observed in the pretreated *Da*_*1*_ and *Dr*_*1*_ samples was reflected in a lower ORC. The values obtained were similar to the ranges reported for *Dioscorea* (Argaw et al. 2023), *Amorphophallus abyssinicus* (Tortoe et al. 2019), and *Solanum tuberosum* (Kolawole et al. 2024) tuber flours. It should be noted that amylose content has also been related to ORC, due to the presence of hydrophobic internal cavities in its structure; however, as in the study of Chiranthika et al. (2022), our findings did not identify a relationship between amylose content and ORC. It has been reported that low oil affinity may be beneficial in the formulation of breaded products (Ferrari and Ferrari 2025). On the other hand, it has been reported that blanching causes starch gelatinization and thus may reduce oil absorption, which offers advantages in frying processes (Jimoh et al. 2024).

#### 3.2.3 Swelling Power and Solubility

The SP of the flours decreased in *Da*_*1*_ and *Dr*_*1*_ flours, being higher in *D. rotundata* flours. The SP values ranged from 4.65 to 11.47 g/g, being within the range reported for *D. alata* (Fawale et al. 2025) but higher than that reported for *D. rotundata* (Tanimola et al. 2022). This behavior suggests a greater interaction between water molecules and starch in *D. rotundata* , in agreement with that reported by Tortoe et al. (2017), who found that the SP of *D. rotundata* was almost 1.5 times higher than that of *D. alata* . In contrast, *D. alata* flours presented low SP due to their high amylose content, which restricts starch expansion by reinforcing the internal network of the granule (Amankwah et al. 2022), maintaining the integrity of the swollen particles (Kumoro et al. 2020; Sahoo et al. 2023). In this sense, amylopectin presents a positive correlation with SP, since it has a branched three‐dimensional structure that provides space for water retention (Chiranthika et al. 2022). According to Sahoo et al. (2023), low swelling power offers advantages against shearing during cooking, which makes the pretreated flours in this study more suitable for processing foods such as noodles. It should be noted that the swelling power of tuber flours has an inverse relationship with their solubility (Varshney et al. 2024).

In line with the above, it was observed that the solubility of *Da*_*1*_ and *Dr*_*1*_ flours increased with the application of pretreatments and the higher proportion of amylose, although without statistically significant differences. Solubility has been related to the degree of amylose leaching from starch granules during swelling (Tortoe et al. 2017). As granules expand, weakly bound amylose is released, which contributes to a higher level of solubility (Fawale et al. 2025). Amankwah et al. (2022) observed that higher solubility coincided with reduced swelling power, which is consistent with that described in the present study.

#### 3.2.4 Dispersibility

Dispersibility is defined as the ability of a flour to reconstitute in water, where higher values indicate better dispersibility (Buhari et al. 2021). High dispersibility implies that particles separate easily, forming a homogeneous mixture, while low dispersibility favors the formation of lumps or sediment. An increase in dispersibility was observed in pretreated *Da*_*1*_ and *Dr*_*1*_ compared with non‐pretreated *Da*_*0*_ and *Dr*_*0*_, in agreement with previous studies in *D. rotundata* (70%–75.2%) (Awoyale et al. 2023) and *D. alata* (61.8%–67.6%) (Obadina et al. 2014). Differences in dispersibility may be attributed to variations in the degree of compactness, as evidenced by bulk density, where higher values favor dispersibility (Oyeyinka et al. 2023). This parameter has also been related to starch content, indicating that there is a positive relationship between these parameters (Buhari et al. 2021).

#### 3.2.5 Foaming Capacity

Foaming capacity (FC) is a functional property closely related to protein content (Srikanth et al. 2023). In line with the data described in Table 2, *D. rotundata* flours presented higher FC than those of *D. alata*. On the other hand, Hu et al. (2018) demonstrated that the FC of dioscorin, the predominant protein in *Dioscorea*, depends on pH, increasing with protein content, and that when pH is above the isoelectric point range, 4.33–5.94 (Kao et al. 2012), its performance is optimized. This was reflected in the higher FC values in samples *Dr*_*1*_ and *Dr*_*0*_ that had higher pH values (Table 1), consequently, the latter, having the highest value and being farther away from the isoelectric point, had the highest FC. The study of Moses and Jeremiah (2019) also reported this behavior; the values reported indicated that *D. rotundata* presented a FC approximately four times higher than *D. alata* .

#### 3.2.6 Emulsion Activity Index

As for the EAI, emulsifying activity index, a decrease was observed in the pretreated flours, *Da*_*1*_ and *Dr*_*1*_, with values in the range of 40%–45%, similar to that reported by Adindu‐Linus et al. (2023) in yam flours 49%–52%, and to that reported by Songuimondenin et al. (2018) in flours of *D. alata* (49.37%) and *D. rotundata* (43.75%). The EAI of water‐soluble polysaccharides is influenced by the presence of proteins, which adsorb at the oil–water interface and form a stabilizing layer around the droplets (Hu et al. 2018; Liu et al. 2018); thus, the higher the protein content in a polysaccharide material, such as yam flour, the higher the emulsion activity. In this sense, the observed decrease in protein content (see Table 2) was reflected in a lower EAI. This functional characteristic is particularly relevant in the preparation of salad dressings (Jenipher et al. 2025).

### 3.3 Thermal Properties

The DSC thermogram of the yam flours is shown in Figure 1b. Endothermic peaks were evident in all samples, although more marked in the *D. alata* flours. Endothermic transitions were present in *Da*_*0*_ and *Da*_*1*_ between 77°C and 82°C, while in *Dr*_*0*_ and *Dr*_*1*_, they were observed in the range of 80°C–85°C.

On the other hand, Table 5 summarizes the parameters describing the thermal properties of *D. alata* and *D. rotundata* flours. The values of To, Tp, and Tc decreased with pretreatments; however, they were within the ranges reported for *D. alata* (Otegbayo et al. 2024) and *D. rotundata* (Argaw et al. 2024). Desai et al. (2024) observed similar behavior in yam flours subjected to blanching and convective drying and attributed this reduction in gelatinization temperatures to the destruction of the crystalline arrangement during blanching, which is consistent with the decrease in the amylopectin portion reported in Table 2, and with that reported by Pachuau et al. (2018) in citric acid‐treated taro starch in which thermal parameters also decreased. In this sense, the decrease in amylopectin leads to less structure to disorganize and thus less need for heat. It was observed that *D. rotundata* flours have higher values of gelatinization temperatures than *D. alata* , indicating that they require more energy to destabilize the amylopectin molecules involved in gelatinization, which is consistent with the order of amylopectin content *Dr*_*0*_ *> Dr*_*1*_ *> Da*_*0*_ *> Da*_*1*_ and with the behavior described in Figure 1b. Furthermore, according to Fawale et al. (2025), a low amylose content is associated with a high gelatinization temperature, which is consistent with our findings.

**TABLE 5:** Thermal properties of *D. alata* and *D. rotundata* flours.

Thermal properties/sample	*D. alata*		*D. rotundata*	
	*Da*_*0*_	*Da*_*1*_	*Dr*_*0*_	*Dr*_*1*_
Onset temperature (T_o_)	76,23 ± 0,41 ^b^	75,89 ± 0,05 ^b^	77,10 ± 0,12 ^a^	77,01 ± 0,09 ^a^
Peak temperature (T_p_)	79,35 ± 0,40 ^c^	78,38 ± 0,42 ^c^	83,79 ± 0,58 ^b^	81,55 ± 0,50 ^a^
Conclusion temperature (T_c_)	89,16 ± 0,29 ^b^	81,56 ± 0,49 ^c^	90,25 ± 0,26 ^a^	90,11 ± 0,33 ^a^
Gelatinization enthalpy (ΔH·J/g peso seco).	13,84 ± 0,05 ^a^	8,60 ± 0,36 ^d^	13,07 ± 0,07 ^b^	11,46 ± 0,19 ^c^

*Note:* Means with the same letter in the same row are not significantly different (*p* < 0.05).

On the other hand, a decrease in the gelatinization enthalpy was evident in the pretreated samples, *Da*_*1*_ and *Dr*_*1*_. This reduction is attributed to the pregelatinization of starch during blanching, which weakened the amylose and amylopectin molecules and reduced the energy required to complete gelatinization (Bora et al. 2023). The decrease in gelatinization enthalpy has also been related to the formation of amylose–lipid complexes (Cervantes‐Ramírez et al. 2020). In agreement with the report of Rodríguez‐Lora et al. (2022), the high enthalpy values in the untreated samples could be attributed to interactions between granule components (amylose‐amylose, amylose‐amylopectin, amylose‐lipid), acting as a thermal barrier and representing a higher amount of heat required for gelatinization. Furthermore, this is consistent with the finding that higher protein content increases the gelatinization temperature by requiring additional energy to destabilize the granule structure, which aligns with the higher protein content in the untreated samples.

In general, there was no gelatinization of the flours at temperatures below 70°C, indicating that these flours do not swell readily and may have potential applications in the manufacture of bakery products where gelatinization at higher temperatures is desired to increase the final volume (Bora et al. 2023).

### 3.4 Thermogravimetric Analysis

Thermogravimetric analysis (TGA) is based on the measurement of the mass loss of the material as a function of temperature to measure the impact of temperature on the composition of the material (Nwadike et al. 2022; Sahoo 2025). Figure 1c illustrates the curves obtained from the thermogravimetric analysis of pretreated and untreated *D. alata* and *D. rotundata* flours.

A similar trend was observed in the TGA curves of all samples of each variety; however, weight loss rates varied with increasing temperature. Three stages of weight loss were identified. The first stage of degradation was observed up to 200°C with a loss of 7%–10%, which corresponded to evaporation of available water and release of low molecular weight volatile material (Martinez et al. 2021); this range is similar to that reported in flours of *D. bulbifera* , *D. pentaphylla* , *D. hispida* , and *D. tomentosa* (9%–11%) (Sahoo et al. 2023) and *D. pentaphylla* (10%–12%) (Sahoo et al. 2025). The migration of volatile material has been associated not only with the increase in temperature but also with the movement of water that generated a dragging action (Hazrati et al. 2021).

The second stage was found between 200°C and 500°C. This region presented the greatest weight loss and corresponded to the degradation of organic compounds such as lipids, proteins, and carbohydrates (Ratnawati et al. 2024) through oxidation and pyrolysis processes (Sahoo 2025). Samples *Da*_*0*_, *Da*_*1*_, *Dr*_*0*_, and *Dr*_*1*_ presented weight loss values of 62.66%, 67.85%, 65.53%, and 67.52%, respectively. The higher mass loss value exhibited by *Dr*_*1*_ could be due to a higher carbohydrate content in this sample. These values were found to be above the ranges reported for porang flours (67.5%), a tuber similar to yam (Ratnawati et al. 2024), and in *D. hispida* (50%–55%) for temperatures between 150°C and 500°C (Sahoo 2025). These data show an increase in weight loss in the pretreated samples, which is consistent with the study of Ratnawati et al. (2024), who reported that treatment with citric acid caused an increase in mass loss by shortening the molecular chains and facilitating their decomposition. This finding coincides with the hydrolysis effect observed in the flour components in the present study. From this perspective, the nonpretreated flours presented the highest thermal resistance. In general, these results suggest that all these flours are thermally stable up to 200°C, which conditions the cooking processes for obtaining derived products.

The third stage was observed at 500°C and exhibited a loss of 10%–12%, due to the formation of inert carbon residues (Martinez et al. 2021), resulting from the decomposition of the products formed during the previous stages due to polymerization processes (Sahoo 2025). The residues were 16.5%, 12.9%, 15.8%, and 12.7% for *Da*_*0*_, *Da*_*1*_, *Dr*_*0*_, and *Dr*_*1*_, respectively, which are consistent with the higher tendency in the pretreated samples to thermal degradation.

### 3.5 Rheological Behavior of Flours

The rheological behavior of the flours, presented in Figure 1d, evidenced an increase in shear stress as the shear rate increased, similar to that observed by Bora et al. (2023). Figure 1e evidenced a decrease in apparent viscosity with increasing shear rate, known as shear thinning (Otegbayo et al. 2024). This behavior has been attributed to the breakdown of the three‐dimensional starch network by mechanical action, which reduces flow resistance (Qian et al. 2019). In general, a rapid increase in shear stress was observed while apparent viscosity decreased drastically, in agreement with that observed by Trinh et al. (2025).

The rheological parameters (Table 6), obtained from the adjustment to the Herschel Bulkley model, indicated flow behavior index values lower than 1 (*n* < 1), characteristic of a shear thinning behavior, which has also been reported in previous studies on flours of *D. rotundata* (Argaw et al. 2024), *D. alata* (Bora et al. 2023), and other varieties such as *D. opposita* (Qian et al. 2019) and *D. esculenta* (Agudelo‐Zamudio et al. 2023). In this context, it can be stated that all flours showed pseudoplastic behavior. This pseudoplasticity has been reflected in products with yam flour or starch, for example, reconstituted enteral formulas (Ferreira et al. 2024), instant soups (Rodriguez‐Lora et al. 2019), and yoghurt (Pérez et al. 2021).

**TABLE 6:** Rheological parameters of *D. alata* and *D. rotundata* flours adjusted to the Herschel Bulkley model.

Parameter/sample	*D. alata*		*D. rotundata*	
	*Da*_*0*_	*Da*_*1*_	*Dr*_*0*_	*Dr*_*1*_
n	0,67 ± 0,01 ^c^	0,59 ± 0,07 ^d^	0,81 ± 0,01 ^a^	0,71 ± 0,07 ^b^
σ_0_ (Pa)	0,89 ± 0,02 ^a^	0,24 ± 0,01 ^b^	0,12 ± 0,01 ^c^	0,11 ± 0,01 ^c^
$K\;\left(\mathrm{Pa}*s\right)$	0,23 ± 0,01 ^b^	0,64 ± 0,02 ^a^	0,05 ± 0,07 ^d^	0,11 ± 0,02 ^c^

*Note:* Means with the same letter in the same row are not significantly different (*p* < 0.05).

A decrease in n values was observed in the pretreated samples of each variety, while the untreated samples exhibited higher n values. When relating this property to swelling power, a similar behavior was evidenced in the study of Bora et al. (2023), who observed a lower susceptibility of granules with lower swelling to shear deformation and disintegration. On the other hand, Oluba et al. (2021) argue that low swelling power in association with high solubility is indicative of stronger associative forces within the flour sample, which is consistent with our findings and justifies the described behavior. Likewise, other studies have related the reduction of apparent viscosity in acid‐treated and blanched samples to the increase of the amylose portion (Agudelo‐Zamudio et al. 2023; Kimbonguila et al. 2019), indicating a higher pseudoplastic tendency, such as those observed in *Da*_*1*_ and *Dr*_*1*_.

On the other hand, the yield stress, σ0, defined as the minimum stress required for a material to begin to flow, exhibited a decreasing trend between the pretreated and non‐pretreated samples of each variety, similar to that reported by Trinh et al. (2025) who observed this trend with increasing periods of acid hydrolysis. On the other hand, the consistency coefficient (K), positively correlated with apparent viscosity (Qian et al. 2019), followed the same trends as this parameter, showing increases in the pretreated samples by the lower swelling powers exhibited. In general, the experimental data evidenced *R* ^2^ values ≥ 0.99, which indicates a high fit to the Herschel Bulkley model and validates the behavior described.

The results of the viscoelasticity analysis of *D. alata* and *D. rotundata* flour samples are presented in Figure 1f–h. The energy absorbed by the flours and recovered after each deformation cycle is represented by the storage or elastic modulus G′, while the energy lost is defined by the loss or viscous modulus G″ (Argaw et al. 2024). According to Figure 1f,g, all flours exhibited a predominance of the elastic properties G' over the viscous G", and an increase of these moduli was observed with increasing angular frequency, similar to that observed in previous investigations (Agudelo‐Zamudio et al. 2023; Bora et al. 2023; Otegbayo et al. 2024). On the other hand, in all samples evaluated, the tangent δ (Figure 1h) was less than unity, indicating that these samples were more elastic than viscous. This result supports the findings on the prevalence of G' over G" and suggests viscoelastic solid behavior, which is consistent with that reported in flours of *D. cayenensis* , *D. bulbifera* , and *D. rotundata* (Argaw et al. 2024).

The following order was observed in the G' modulus, *Da*_*0*_ *> Da*_*1*_ *> Dr*_*1*_ *> Dr*_*0*_. The lower G' of *D. rotundata* flours could be attributed to a lower structural integrity of the granules, as a result of a higher swelling power (Bora et al. 2023; Otegbayo et al. 2024). Consequently, the lower G' values evidenced in *D. alata* are associated with a lower degree of swelling, which gives these flours greater elasticity. In particular, the *Da*_*0*_ sample is the most appropriate for processes that require materials with high resistance to deformation, which makes this flour a possible option for the production of doughs for noodles and bread.

However, when analyzing the effect of the pretreatments on the G' modulus, a significant increase was observed in the treated samples (*Da*_*1*_ and *Dr*_*1*_), compared to the controls (*Da*_*0*_ and *Dr*_*0*_), which suggests improvements in rheological behavior by conferring greater mechanical strength properties. On the other hand, the potential to form less viscous and more elastic gels and masses exhibited by the flours, *Da*_*0*_ and *Da*_*1*_, could make them more suitable as thickeners for sauces and soups, and as binders for meat products (Oluba et al. 2021).

#### 3.5.1 Pasting Properties

When heat is applied to starch‐rich foods, such as flours, in the presence of water, gelatinization processes are triggered that involve important transformations (Babalola et al. 2021), such as those shown in Figure 1i, which are fundamental for predicting the behavior of flours in food processing (Argaw et al. 2024; Asranudin et al. 2023).

The summarized pasting parameters are presented in Table 7 and are consistent with the findings shown in Figure 1i. Lower pasting temperatures were observed in the pretreated flours. Similar results were reported by Argaw et al. (2024) in *D. rotundata* and by Asranudin et al. (2023) in *D. alata* . Pasting temperature indicates the change in viscosity due to the swelling properties of starches (Danso et al. 2025). In this sense, a high pasting temperature indicates resistant starch, so differences in pasting temperature between flours could be related to granule size and amylose content (Argaw et al. 2024). Generally, higher pasting temperatures suggest stronger binding and smaller granule sizes, leading to higher rupture resistance (Fawale et al. 2025). This coincides with the lower particle sizes and amylose contents presented by the non‐pretreated samples, as well as the higher amylopectin content observed in the DSC thermogram. This results in a higher temperature requirement for complete gelatinization.

**TABLE 7:** Pasting properties of *D. alata* and *D. rotundata* flours.

Pasting properties/sample	*D. alata*		*D. rotundata*	
	*Da*_*0*_	*Da*_*1*_	*Dr*_*0*_	*Dr*_*1*_
Pasting temperature (°C)	79,52 ± 0,29 ^b^	77,97 ± 0,34 ^c^	82,69 ± 0,11 ^a^	77,07 ± 0,27 ^d^
Peak time (min)	7,62 ± 0,14 ^b^	7,14 ± 0,07 ^c^	6,82 ± 0,09 ^d^	9,44 ± 0,09 ^a^
Peak viscosity (cP)	11,346,84 ± 3,42 ^c^	10,506,51 ± 3,43 ^d^	26,296,95 ± 5,00 ^a^	23,886,79 ± 4,89 ^b^
Trough viscosity (cP)	4615,156 ± 4,11 ^d^	5336,743 ± 3,99 ^b^	6196,39 ± 3,98 ^a^	4990,45 ± 4,49 ^c^
Breakdown (cP)	6731,68 ± 1,75 ^c^	5169,77 ± 0,55 ^d^	20,100,56 ± 1,03 ^a^	18,896,33 ± 1,24 ^b^
Final viscosity (cP)	5087,20 ± 4,21 ^d^	6091,44 ± 4,64 ^b^	6871,54 ± 4,26 ^a^	5148,74 ± 4,61 ^c^
Setback (cP)	472,05 ± 1,65 ^c^	754,70 ± 2,46 ^a^	675,15 ± 0,36 ^b^	158,28 ± 1,15 ^d^

*Note:* Means with the same letter in the same row are not significantly different (*p* < 0.05).

On the other hand, the high pasting temperatures exhibited by *Da*_*0*_ and *Dr*_*0*_ suggest that they do not form paste easily and, being resistant to heating, can be used as heat‐treatment–resistant thickeners and foods intended for sterilization (Matsumoto et al. 2021).

On the other hand, the peak viscosity values of the samples decreased in the samples with pretreatment, *Da*_*1*_ and *Dr*_*1*_. *D. rotundata* flours presented the highest peak viscosity values, highlighting *Dr*_*0*_ with the highest value, while *D. alata* samples exhibited lower values, with *Da*_*1*_ having the lowest value, a behavior similar to that observed by Pérez et al. (2021) who observed that *D. rotundata* starches had more capacity to generate gels with higher viscosity than *D. alata* . Previous studies reported that hydrothermal treatments, such as blanching, in combination with acid hydrolysis treatments, such as critical acid treatment, decrease the pasting properties (Asranudin et al. 2023). This trend was observed in Figure 1i. In addition, the curves described an initial phase with a gradual increase in viscosity as the temperature increased; this phenomenon has been attributed to swelling of the granules. It was recently established that there is a positive correlation between swelling power and peak viscosity; furthermore, the literature indicates that a higher amylose content inhibits starch swelling and reduces viscosity (Fawale et al. 2025; Sahoo et al. 2023) this behavior was reflected in our results where samples high in amylose had lower peak viscosity values, while samples categorized with intermediate amylose exhibited higher peak viscosity values.

On the other hand, a decrease in the viscosity of the flours was identified in the isothermal temperature period (95°C), which corresponded to the minimum viscosity of all samples. Similarly, a decrease in the breakdown was observed in the pretreated flours. A similar behavior was observed by Asranudin et al. (2023) and was associated with the strengthening of the granule, which caused it to repel disintegration due to a reinforcement of intragranular bonding forces after pretreatments, which is also consistent with a lower swelling and higher pasting temperature. It should be noted that the trough viscosity behavior was different among the varieties. An increase was observed in *D. alata* and a decrease in *D. rotundata* , which may be associated with interactions of starch with other components, such as ash (William et al. 2023) and lipids (Huang, Chiu, et al. 2024) that may interfere with pasting properties.

In the cooling period from 95°C to 50°C, corresponding to the final viscosity and setback, an increase in viscosity was observed in all samples due to molecular reassociation. The trend between treated and pretreated samples was the same as that described for trough viscosity. William et al. (2023) evaluated flours from different sources and found that blanching increased the final viscosity and setback in some samples, while in others it decreased. Setback, an indicator of the tendency to retrogradation, has been related to the hardening of bread. It was observed that *Da*_*1*_ presented the highest setback, which agrees with the finding of Tortoe et al. (2017) who reported a higher tendency to retrograde in flours of *D. alata* than *D. rotundata* .

## 4 Conclusions

Overall, current research has laid a solid foundation for understanding how immersion, blanching, and soaking processing methods affect the properties of yam flours from the *D. alata* and *D. rotundata* varieties. These flours responded similarly to pretreatments, with variations in the magnitude of the response. The pretreatments showed a slight decrease in nutritional content; however, at the functional level, they demonstrated improvements in properties such as water retention capacity, solubility, and dispersibility. They also significantly modified the thermal and rheological properties of the flours, resulting in flours with lower gelatinization temperatures, which implies lower energy consumption during processing, as well as greater shear resistance, giving them greater rheological stability.

No single flour was identified as “best” in terms of variety, as all flours exhibited characteristics that allow for their potential use in a wide range of food products. These multifaceted differences provide relevant information for food technologists and manufacturers and enable decisions to be made about flour selection and optimization based on process and product requirements.

Future research should explore in greater detail the structural changes induced by pretreatments through the study of granule morphology, changes in nutrient bioavailability, and evaluation of micronutrient content, in order to understand in greater detail the changes generated and identify other possible applications in the food industry and other areas.

## Author Contributions

Piedad Montero Castillo contributed to conceptualization, investigation, formal analysis, supervision, resources, project administration, and original draft writing; Diofanor Acevedo Correa contributed to investigation, supervision, visualization, and manuscript review and editing; and Karina Vivanco Zuñiga contributed to methodology, data curation, and manuscript review and editing.

## Funding

This work was funded by the Sistema General de Regalías de Colombia (SGR), through the project identified with the code BPIN‐2020000100439, beneficiary of the Call 006–2019 for eligible research and development projects.

## Ethics Statement

The conduct of this research did not involve the participation of humans or animals in the collection of data or in the performance of experiments. All data used were publicly available and did not involve any ethical risk.

## Conflicts of Interest

The authors declare no conflicts of interest.

## Data Availability

The data that support the findings of this study are available from the corresponding author upon reasonable request.
